# Farmers Knowledge, Attitudes, Practices and Health Problems Associated with Pesticide Use in Rural Irrigation Villages, Southwest Ethiopia

**DOI:** 10.1371/journal.pone.0162527

**Published:** 2016-09-13

**Authors:** Hailay Abrha Gesesew, Kifle Woldemichael, Desalegn Massa, Lillian Mwanri

**Affiliations:** 1 Department of Epidemiology, Jimma University, Jimma, Ethiopia; 2 Discipline of Public Health, Faculty of Medicine, Nursing, and Health Sciences, Flinders University, Adelaide, Australia; Universiteit Gent, BELGIUM

## Abstract

**Background:**

In Ethiopia, pesticides are widely used for a variety of purposes. The occurrence of contamination and poisoning for farmers is highly reported due to unsafe handling practices and their usage. We assessed knowledge, attitudes and experiences of previous pesticide exposure, and related health problems among farmers who use irrigation in Jimma Zone, Southwest Ethiopia.

**Methods:**

A community based cross-sectional study was conducted among farmers living in the zone. Respondents were 796 irrigation farmers from 20 kebeles (lowest administration unit) in Jimma Zone. Data were collected using a pretested and structured questionnaire via face-to-face interviews. Both descriptive and inferential statistics analysis were performed. A binary logistic regression was used to identify factors associated with attitudes of farmers towards the safe use of pesticides at P value of ≤ 0.05 in the final model.

**Results:**

Among the participants, 54.4% (95%CI, 50.7–58%) knew at least one pesticide control method and 53.7% had positive attitudes towards safe use of pesticide. The mean score of attitudes was found to be 3.9(±0.4). Knowledge including each of the following: the names of the pesticides (AOR, 0.41; 95%CI, 0.25–0.67), methods of pest control and the use of gloves during pesticide exposure (AOR, 1.52; 95%CI, 1.07–2.16) was found to be independent predictor of the farmers’ attitudes about safe use of pesticides. Past exposure of pesticide was reported by 89.6% of farmers. Participants reported ingestion (88.9%) and inhalation (90.4%) as possible mechanisms of pesticide exposure. Nearly 42% of farmers had never used any personal protective equipment (PPE) to protect themselves against pesticide exposure. Farmers reported several health complications, which were perceived as complications of pesticide exposure, including: headache, nausea and vomiting, skin rash and irritation and abdominal pain.

**Conclusions:**

The study exposed the existence of high probability of pesticide exposure, the low safe use of pesticide and the low use of PPE. However, but farmers had positive attitudes towards safe use of pesticides. These findings appeal for the development of effective public health strategies to improve farmers’ awareness and safe use of PPE. In addition, there is a need to inform farmers about integrated pest management to prevent severe health complications, which may occur as a result of unsafe and inappropriate use of pesticides.

## Background

Evidence exists of unnecessary and unacceptable occurrences of high level of contamination and poisoning of pesticide users, agricultural workers and bystanders across the world[1]. In recent years these have been pressing public health and food safety concerns related to pesticide residues. Increased reporting of these problems may partially be related to growing consumer demand for safe food, not only in developed countries, but also increasingly in developing countries. Human exposure to pesticides occurs primarily through dietary residues, outdoor pesticide exposures, indoor pesticide exposures, occupational exposures, and through unsafe use of pesticides on domestic animals[2]. Furthermore, there are concerns about contamination of drinking water sources with pesticides or their by-products.

Worldwide, a significant number of people are deceased annually from the consequences of pesticide exposure [3,4]. Short-term complications such as acute pesticide poisoning have been reported as major consequence in the farming community [5]. There are reported long term health effects, including carcinogenic and endocrine disrupting properties, especially on vulnerable groups [1]. According to previous studies, factors contributing for the morbidity and mortality of pesticide exposure included inadequate knowledge [6,7], non-use or inappropriate use of PPE [8–10], improper storage of pesticides at home [5,9,11], negative attitude towards pesticide [7], and inappropriate practices [10]. A study conducted on pesticide use on agricultural farms in Ethiopia, illustrated that the sprayers accepted the recommended washing if pesticides splashed on their bodies but did not seem convinced of the benefit of going to a health clinic to receive further treatment. A few believed that working with pesticides should not be a problem at all, while the vast majority considered careful handing to be more important than using PPE [10].

All pesticides have the potential to harm human, animals, or other living organisms and the environment if used incorrectly [12]. A study from Tanzania revealed that 68% of farmers reported several health symptoms inducing feeling sick, skin problems and neurological symptoms [13] following exposure to pesticides. Several strategies have been recommended as key to preventing pesticide exposure to farmers. These include: having correct knowledge and attitudes about protection against pesticides, safe practices, such as effective use of PPE, and understanding of labels. We assessed knowledge, attitudes and experiences of previous pesticides exposure, and related health problems among irrigation farmers in rural setting, Southwest Ethiopia.

## Materials and Methods

### Study design, setting and participants

A community based cross–sectional study was conducted between March 15 and 30, 2014 in Jimma zone, Southwest Ethiopia. Jimma Zone is located 357km southwest of Addis Ababa, the capital city of Ethiopia. According to the 2007 census, the zone has a total population of 2,486,155 of which 89.69% are rural inhabitants [14]. It has 17 districts and one administration town with a total of 545 Kebeles (the smallest administrative unit) of which 515 are rural. An average of 4.8 persons to a household (3.8 in urban and 5 in rural households) are residing in the zone [14]. The commonest crops in Southwest Ethipia are coffee, grains, ‘*khat’*, sorghum and ‘*enset’* (false banana). The study was conducted in five districts with high irrigation sites (access to irrigation canals) in 20 kebeles namely, Nadda Chala, Nadda Dawa, Burka Asendabo, Waktola, Asendabo, Birbirsa Waritu, Tijje Koye, Bakke Agalo, Tamsa Jida, Tullu Kidida, Gejjib, Maro Chisa, Dora, Seka, Gibe Bosso, Gurra Ulauke, Atro Sufa, Shashemanne and Meti. Head of households were the respondents.

### Sampling

Multistage sampling technique was used to recruit the participants. Five districts with high coverage of irrigation were selected. From each district,four Kebeles with high coverage of irrigation were chosen. A total of20 kebeles were selected for the study. The sample population per kebele were allocated via proportional to population size (PPS). The sample population were selected from each kebele using simple random sampling technique by taking sampling frame from agriculture sector records. The required sample size was calculated using single population proportion calculation formula and the following assumptions: 55.9% proportion of farmers who were appropriately provided with respirator when spraying pesticides [10], 95% confidence level, 5% margin of error, 80% power, and 5% estimated non–response rate. Considering a design effect of 2, the calculated sample farmers were totalled to be 796.

### Data collection

We scheduled a face-to-face interview with the head of the households to request the aforementioned information. A total of ten data collectors and three supervisors who had previous survey experience were trained on the aim, of the importance of confidentiality of information, respondent’s right and procedures of interview prior to data collection. Data were collected using a pretested and structured questionnaire. The questionnaire was pretested among farmers living near the study setting, and necessary corrections were made. Participants were interviewed at their home. We gathered information on socio- demographic and economic characteristics of respondents, pesticides names, pesticide exposure routes, pesticide control methods, actions that were taken after poisoning, storage and disposal, PPEs, attitudes about hazardous effect of pesticides, practices of farmers for pesticides application and health problems related to pesticides.

The farmers were asked to report the pesticides by trade names or local names. Data collectors checked the name of pesticide from the container or label when farmers failed to do so. The corresponding active ingredients were accessed from the national list of registered pesticides[15]. Self reported post pesticide exposure symptoms were also collected. Data collectors checked pesticide storage and PPE availability for those who reported having pesticides in storage during data collection period. However, when farmers reported that they had used pesticide but did not have the container or containers, data were based on their report.

Farmers’ attitudes about pesticides use and their adverse effect on human health were assessed using a likert scale based tool consisting of nine items, which had five ranges each (strongly disagree = 1, disagree = 2, undecided = 3, agree = 4 and strongly agree = 5). The nine items included: i) Most pesticides create some risk of harm to humans, animals, or the environment; ii) Most pesticides exposures happen not in the workplaces, but through foods, and in the home or garden; iii) It is very likely for pesticide residues to be present inside or on the surfaces of the foods we eat and water we drink; iv) Reuse of empty pesticide containers predisposes to pesticides exposure and poisoning; v) Personal protective equipment (PPE) use can reduce pesticides exposure and poisoning; vi) Eating, chewing or drinking in the sprayed agricultural field increases pesticides exposure and poisoning; vii) Pregnant women handling pesticides at first trimester have greater health risk and as well to the fetus; viii) Children of farming families are at greater risk of pesticide exposure and poisoning; and ix) Disposing of pesticides properly can help to protect the environment. Attitude score was calculated via computing mean of sum of the ranges.

### Data analysis

We entered data into EpiData version 3.1 and exported into SPSS version 21 software for analyses. Descriptive statistics included mean, median, standard deviations, and range values for continuous data; percentage and frequency tables for categorical data. Bivariate logistic regression analysis was conducted to see the existence of crude association. Selected candidate variables (with P value below 0.25) were considered to multivariable logistic regression. We checked multi-collinearity among selected independent variables via variance inflation factor (VIF) and none was found. P-value ≤ 0.05 was considered as a cutoff value for statistical significance in the final model. Goodness of fit of the final model was checked by Hosmer and Lemeshow and was found fit. The data were summarized using odds ratio and 95% confidence interval.

### Ethical statement

The study was approved by institutional review board (IRB) of college of health sciences at Jimma University, Southwest Ethiopia. Permission for the study to be conducted was also obtained from the zones, districts and kebeles. Participants received explanations of the study and of the purpose of the study in their mother tongue. Informed consent was obtained from study participants before the commencement of each interview, and no personal identification was registered. We prepared an informed verbal consent that involved purpose of the research, expected duration of the interview, and a description that the participants could withdraw from the interview at any time, had no risk and no payment for their recruitment. We read this statement to each study participants before conducting the interview and requested their permission to be involved in the study.

We proposed a verbal consent over written consent for the following reasons: i) This was cross-sectional study that enquired descriptive data; ii) Their responses had no personal, social or political consequences; iii) We believed that there would not be significant risks to the participants; and iv) A significant number of farmers in Ethiopia have no educational status. The IRB approved the proposed verbal consent procedure. The Confidentiality of the data was ensured and access to raw data was allowed only after a joint agreement by the investigators involved in designing, conducting and financing the study.

## Results

### Characteristics of the study participants

In total, 719 farmers responded to the study making a response rate of 90.3%. Non-participation was because of interview refusals (4.8%) and closed houses (5.2%). Table 1 shows characteristics of the respondents. Males were over-represented (67.3%) and most of the study participants were aged between 21–30 years (32.4%). About two third (67.5%) of participants could read and understand labels/instructions from the pesticide container if written in the local language. A significant proportion (63.4%) of farmers had children in their household and 23.5% households had pregnant or lactating women. Out of the 719 farmers; 461(64.1%), 225(31.3%) and 192(26.7%) cultivated cereals, fruits and vegetables respectively. Around 66% of the farmers reported *khat* chewing and 17.7% cigarette smoking.

**Table 1:** Characteristics of farmers.

Variables		Frequency	%
**Sex**	Male	484	67.3
	Female	235	32.7
**Age in years**	<20	54	7.5
	21–30	233	32.4
	31–40	179	24.9
	41–50	134	18.6
	51–60	68	9.5
	>60	51	7.1
**Educational status**	Illiterate	234	32.5
	Able to Read and write only	41	5.7
	Literate (formal education)	444	61.8
**Presence of children in the HH**	Yes	456	63.4
	No	263	36.6
**Presence of pregnant/lactating mother in the HH**	Yes	169	23.5
	No	550	76.5
**Cultivated Agricultural Products** ^**a**^	Cereals and legumes	487	65.7
	Fruits and vegetables	417	58.0
	Cash crops(*khat*, coffee and inset)	92	12.8
**Cigarette smoking**	Yes	127	17.7
	No	592	82.3
**Chat chewing**	Yes	471	65.5
	No	248	34.5

^a^ Farmers reported more than one product

### Farmers’ knowledge, attitudes, practices and health problems associated with pesticide exposure

Table 2 shows knowledge of the farmers towards safe use of pesticides. a significant proportion (87.2%,95%CI, 84.8–89.6%) of participants knew pesticides by name. Ingestion (88.9%) and inhalation (90.4%) were the major reported routes of pesticide exposure. Three hundred ninety one (, 54.4%, 95%CI, 50.7–58%), farmers knew at least one of the following pest control methods: manual removal, using bed-net and applying smoke. The majority (87.5%) of participants knew at least one health problem on humans following exposure to pesticides. Only 45(6.3%) participants knew possible health problems in pregnancy following pesticide exposure. Congenital malformation (29, 4.1%) and prenatal death (16,2.2%) were reported as possible outcomes of pregnancy following exposure to pesticides. More than half of participants (53.8%) had knowledge on negative effects of pesticide on animal health. More than half (55.2%) of the participants did not agree that most pesticides exposures happen in the workplaces, but through foods in the home or garden. Thirty-eight precent of the participants did not agree with the statement that reads, “it is very likely for pesticide residues to be present inside or on the surfaces of the foods we eat and water we drink”. The mean score of attitudes was found to be 3.9 (±0.4). Over half of participants (386, 53.7%) had positive attitudes towards safe use of pesticides.

**Table 2:** Knowledge of farmers towards safe use of pesticides.

Variables		Frequency	%
**Know pesticide/s by name**	Yes	627	87.2
	No	92	12.8
**Pesticide control methods** ^**a**^	I do not know	328	45.6
	Manual removal	210	29.2
	Use bed-net	142	19.8
	Personal and environmental Hygiene	63	8.8
	Apply smoke	33	4.6
	Others (biological, expose to sunlight)	23	3.2
**Routes of pesticide exposure** ^**a**^	Inhalation	650	90.4
	Ingestion	639	88.9
	Skin	252	35.0
	Others	6	0.8
**Problems related to pesticide exposure on human health** ^**a**^	Death	490	68.2
	Pneumonia	329	45.8
	Skin rash	246	34.2
	Asthma	170	23.6
	Do not know	90	12.5
	Congenital malformation	29	4.0
	Prenatal death	16	2.2
**Life time symptoms associated with pesticide exposure** ^**a**^	Headache	17	
	Nausea and vomiting	13	
	Skin rash and irritation	13	
	Abdominal pain	12	
	Los of consciousness	7	
	Cough	7	
	Eye irritation and redness	7	
	Shortness of breath	4	
	Bleeding	3	
	Fever	2	
	Others ^b^	3	
**Effect of pesticide exposure on animal health**	Yes	387	53.8
	No	332	46.2
**Effect of pesticide exposure on the environment**	Yes	89	12.4
	No	630	87.6
**Most common route of exposure**	Inhalation	503	69.9
	Ingestion	169	23.5
	Skin	35	4.9
	Missing	12	1.7
**Most dangerous route of exposure**	Ingestion	462	64.2
	Inhalation	194	26.9
	All are equally dangerous	23	3.2
	Skin	12	1.7
	Missing	28	3.9
**Pesticide residuals exist in** ^**a**^	Air	580	80.7
	Soil	183	53.5
	Water	140	19.5
	Cereals/legumes	119	16.6
	Vegetables	105	14.6
	Fruit	94	13.1
**Personal Protective Equipment types** ^**a**^	Mask	397	55.2
	Special boot	272	37.8
	Glove	252	35.0
	Wide brimmed hat	186	25.9
	Goggle	113	15.7

^a^ more than one answer was reported (not mutually exclusive)

^b^ Others included: sweating, throat pain and chest pain

Table 3 shows farmers’ practices towards the safe use of pesticides. Six hundred and forty four participants (89.6%) had ever used pesticides. Out of the farmers who had ever used pesticides, (599, 93.0%) were current users and (526,81.7%) had used pesticides for more than three years at the time of this study. From the farmers who had ever used pesticides, 407 (63.2%) usually followed the instructions/labels written on the containers of the pesticides. Two out of five farmers (41.8%) reported using no PPE at all. More than half (58.2%) of participants reported to have usually used at least one PPE. Locally prepared mask (39.9%), boot (29.4%) and hat (21.1%) were most often used PPE. The mean time taken to re-enter to their work/agricultural field after pesticide spray was found to be 9.7 hours. Less than half (46.3%) of respondents disposed empty pesticide containers, and 12.6% reused them for storage of food items in the household.

**Table 3:** Practice farmers towards safe use of pesticides.

Variables		Frequency	%
**Ever used pesticides**	Yes	644	89.6
	No	75	10.4
**Currently using pesticides**	Yes	599	83.3
	No	120	16.7
**Duration of pesticide use**	3 years and below	120	18.6
	4–10 years	327	50.8
	Above 10 years	182	28.3
	Do not know	15	2.3
**Follow the instruction on pesticides bottle's label**	Yes	407	63.2
	No	237	36.8
**Empty pesticide containers**	Burning, burring or put to toilet	298	46.3
	Throw it on the garbage sites or streets	178	27.6
	Used for storage of food items	81	12.6
	Used storage for other pesticide	71	11.0
	Others (used for storage of non-food items like kerosene)	16	2.5
**PPE use during pesticide** ^**a**^	Locally prepared mask	257	39.9
	Boot	189	29.4
	Hat/cap	136	21.1
	Glove	121	18.8
	Goggle	43	6.7
**During spraying pesticide**	Nothing	629	87.5
	Chew *khat*	49	7.6
	Drink/eat food	23	3.6
	Smoke cigarette	18	2.8
**Taking shower immediately after finishing spraying**	Not at all	164	25.5
	Some times	220	34.2
	Always	260	40.3
**Pesticide exposure toxicity in family**	Yes	63	9.8
	No	581	90.2

^a^ Farmers reported more than one PPE type

Table 4 shows about present pesticides reported to be used by farmers. The pesticides most commonly reported by farmers as associated with poisoning were DDT (21.9%), diatomaceous earth (12.1%), Malathion (9.9%) and the mixture DDT and diatomaceous earth (12.6%). Of the reported pesticide and associated with poisoning, 41.8, 31 and 24.7% were organochlorine, organophosphate and inorganic respectively (not mutually exclusive), and 69% were moderately toxic products (WHO Class II).

**Table 4:** Pesticides reported as used by farmers in rural irrigation villages.

Pesticide name	Chemical group	WHO class	Frequency, n (%) (n = 644)
**Malathion (diethyl (dimethoxy thiophosphorylthio)**	Organophosphate	III	64 (9.9)
**DDT (1,1,1-trichloro-2, 2-di(4-chlorophenyl)ethane)**	Organochlorine	II	141 (21.9)
**Diatomaceous earth (silicon dioxide)**	Inorganic	III	78 (12.1)
**Tricarnam (Carbaryl Propoxur)**	Organic Carbamates	II	47 (7.3)
**Roundup (N-(phosphonomethyl)glycine) **	Organophosphate	II	59 (9.2)
**Dacamine (2,4-Dichlorophenoxyacetic acid (2,4 D))**	Organochlorine	II	39 (6.1)
**Malathion and DDT**	Organophosphate and Organochlorine	II	69 (10.7)
**Malathion and 2,4 D**	Organophosphate and Organochlorine	II	8 (1.2)
**DDT and Diatomaceous earth**	Organochlorine and Inorganic	II, III	81 (12.6)
**Unknown** ^**a**^	——-	——	41 (9)

^a^ Farmers reported that they used pesticide but neither know the name of pesticide nor showed its container

Among the farmers who had ever used pesticide, 63 (9.8%) reported to have experienced varying forms of health problems following pesticide exposure. The commonest reported symptoms experienced by the victims included headache (n = 17), nausea and vomiting (n = 13), skin rash and irritation (n = 13) and abdominal pain (n = 12) (Table 2). Eight out of 63 (12%) participants reported pesticide toxicity related deaths in the family. Most farmers (n = 28) managed the symptoms via home-based care that included drinking milk, applying local creams on the affected area and washing the affected area. One death was reported during home-based care. Out of the victims to pesticide toxicity, only 13(20.6%) farmers reported seeking care from a health facility. Among those who sought care from the health facility, two cases passed away in the health institution. However, 16 victims did nothing and five deaths were reported.

### Factors associated with attitudes of farmers towards safe use of pesticides

Several factors were associated with farmers’ attitudes towards safe use of pesticides on bivariate analysis model. These included: sex, educational status, knowledge about the name of the pesticides, awareness on pesticides’ adverse effect on environment, awareness that the use of glove can reduce pesticide exposure and knowing that the use of a hat can reduce pesticide exposure. On the multiple binary logistic regression analysis (Table 5), knowledge including each of the following: the names of the pesticides (AOR, 0.41; 95%CI, 0.25–0.67), methods of pest control and the use of gloves during pesticide exposure (AOR, 1.52; 95%CI, 1.07–2.16) was significantly associated with farmers’ attitudes towards safe use of pesticides. The likelihood of having positive attitudes among farmers who didn’t know pesticides by their name was lower than those who knew pesticides by their name (AOR 0.41, 95% CI, 0.25–0.67). Positive attitudes towards safe use of pesticides among farmers, who were aware of reduction of pesticide exposure by using glove, was 1.52 times higher than those farmers who didn’t have awareness (AOR 1.52, 95% CI, 1.07–2.16).

**Table 5:** Factors associated with attitudes of farmers towards safe use of pesticides.

Factors		Attitude		Crude		Adjusted	
		Positive	Negative	OR	95% CI	OR	95% CI
**Sex**	Male	215	269	1.26	0.92,1.72	1.35	0.98,1.87
	Female	118	117	1		1	
**Educational status**	Illiterate	116	159	1.31	0.97,1.77	1.30	0.95,1.78
	Literate	217	227	1		1	
**Know pesticide by name**	Yes	305	322	0.46	0.29,0.74	0.41	0.25,0.67^a^
	No	28	64	1		1	
**Aware of pesticide’s adverse effect on environment**	Yes	47	42	0.74	0.48,1.16	0.78	0.49,1.24
	No	286	344	1		1	
**Know use of glove can reduce pesticide exposure**	Yes	113	141	1.20	0.89,1.61	1.52	1.07,2.16^a^
	No	220	245	1		1	
**Know use of hat can reduce pesticide exposure**	Yes	95	91	0.77	0.55,1.08	0.70	0.48,1.01
	No	238	295	1		1	
**Know use of mask can reduce pesticide exposure**	Yes	197	200	0.74	0.55,0,99	0.73	0.53,1.01
	No	136	186	1		1	

^a^ Statistically significant variables with P-value of <0.05

## Discussion

The issue of knowledge, attitudes and practices about pesticide usage and related health problems among irrigation farmers has been left neglected in Ethiopia. In this study, the majority of farmers were aware about the route of pesticide exposure including inhalation and ingestion, a finding consistent with other studies [16–18]. However, only a small proportion of farmers were aware that skin was one of routes of exposure to pesticides toxicity unlike the finding from the Tanzanian study that reported awareness about the dermal exposure route in 75.2% of farmers [16,18]. Farmers poor knowledge about different exposure mechanisms is critical because skin is the most common route of exposure to pesticide toxicity and famers should know this in order to protect themselves [13,19,20]. While the majority of the farmers were knowledgeable about adverse effect of pesticides and related health problems including on respiratory system and gastrointestinal tract, which was also findings recorded from a previously conducted study in Ethiopia[21], only a few (6.2%) of participants in the current study knew the effect of pesticides in pregnancy. This is an important shortfall in knowledge among participants, as it is necessary to protect pregnant women and unborn babies from pesticide toxicity [17,20].

When compared to a similar study conducted in Palestine that showed that 85.8 and 57.7% of the participants had awareness about adverse effect of pesticides on livestock health and environment respectively [22], the awareness of farmers related to the effect of pesticides on these parameters was lower in the current study. Similarly, a study from Brazil also reported in contrast to the current finding [23]. The variation of awareness between these two studies could be due to the differences in the educational levels of the study participants as there was better literacy level among participants in Palestine and Brazil [22,23]. Consistent with a study in the Gaza Strip [24], the current study depicted that more than half of the current study participants knew about the existence of pesticide residues in air and soil, and below half of respondents acknowledged the existence of pesticide residues in water. However, there were differentials in knowledge about the existence of pesticide residues in vegetables between participants of the current and Gaza studies, with higher percentage of knowledge about this in respondents from the Gaza strip study. The work on improving awareness creation on the harmful effects and prevention strategies associated with pesticide exposure should be taken as the main task because farmers dwelling on most farms are located near the field [23,25,26]. This means that ooccupational health and safety should be integrated in agricultural health care.

The mean score of attitudes was found to be 3.9 (±0.4) and only around half of them had positive attitudes (scored above the mean) towards safe use of pesticides. A study conducted among Thai farmers revealed that about 69.3% of participants had positive attitudes [27]. Of significant interest in the current study, only a small proportion (12.6%) of farmers used the empty containers of pesticides for storage of food items in the household. This is different from the previous study conducted in Ethiopia [21] that reported 77.2% of farmers using empty containers for other purposes. However, the improper disposal/use of empty pesticides containers might expose humans, animals and environment to pesticide toxicity and this needs to be addressed in order to improve farmers’ quality of life. The farmers in the current study also reported disposing empty containers in the streets (28%) while others used pesticide containers to store food or non-food items (26%). This is very serious and therefore, opportunities to safely discard and recycle empty containers should be developed.

The storage of the aforementioned pesticides in home implied the possible exposure to the farmers and their family members. Some of the pesticides are either banned in the country such as DDT or are moderately hazardous such as Malathion. However, WHO class I pesticides were not reported as a cause for health related problem which might be due to the fact that these are registered for “restricted use” [16]. Organochlorine and organophosphate were the major reported chemical groups associated with poisoning and this is consistent with findings of other studies [7,16,18,28]. This study also found that actions taken to address pesticide exposure poisoning were unsatisfactory. Only a few of participants reported to have visited health institutions after pesticide exposure, and others undertook home based care or did nothing. Similar findings were reported by the study conducted in Tanzania [16]. Several reasons could explain such behaviours including: the cost of health care, poor access to services that mitigate pesticide exposure and poisoning, being unaware of symptoms and perceived unavailability of treatment [16]. The introduction of bio-pesticides i.e. the use of bio-control agents (bacteria, fungi and viruses) should extensively be familiarized among farmers, as bio-control agents are safe to human, environment and livestock [29]. In addition, the government should design strategies that can reduce pesticide exposure of farmers and the general population such as restricting hazardous pesticides, monitoring tags, and enforcing good agricultural practices [28]. Empowering economy to make personal and environmental hygiene feasible could also be another strategy [16].

Despite the fact that the majority of the farmers had average knowledge on the possible problem of pesticide exposure, substantial proportion of farmers (41.8%) reported to have never used any PPE. The use of PPE in our study was much lower when compared to findings from a study carried out in Ethiopia that reported 100% of the study participants used PPE[30]. Our findings demonstrated lower indices of desired outcomes when compared with findings from international studies such as a study conducted in East Jamaica [31] and Brazil [23]. The differences in awareness of the route of exposure to pesticides, the poor availability of protective gears in Jimma Zone could be the plausible reason of differences [23,32]. Similar to other studies examining side effects of pesticide to human health, the findings of this study revealed serious and fatal consequences of pesticide exposure to human health when inadequate protection was exercised [33]. Although fatal consequences were rare, many studies have illustrated other subtle but serious short term health problems which over time could lead to chronic ill health with significant social and economic impact on affected individuals, families and communities [31].

There are worth noting limitations in the current study, however. People may only remember one name of pesticide or did not know the name of the pesticide and therefore could report false answers. This self-report of pesticides may under or over report the proportion of pesticides use. Because a significant number (32.5%) of farmers were illiterate, information bias could also have been the case. The duration of pesticide use varied from below three years to above 10 years. This difference in duration of pesticide exposure could have been associated with under reporting of the number of victims who visited health institutions and the type of symptoms due to the fact that long-term complications, mostly, needs cumulative exposure. Some of the symptoms of pesticides intoxication are similar to symptoms resulting from other common diseases and thus, the findings should be interpreted with caution. In addition, estimates by WHO class could also be misinterpreted. This has an impact especially when single active ingredients have been evaluated.

## Conclusions

In summary, the findings from the current study agree in many points with the findings of previous publications, including a high potential for pesticide exposure and pesticide toxicity. The majority of the farmers were aware about the routes of pesticides exposure including that exposure occurs through ingestion and inhalation. However, there is a lack of awareness regarding the dermal route of exposure to pesticide, effect of pesticide on pregnancy and the effect of pesticides on animal health and environment. Although farmers were found to have positive attitudes towards the harmful effect of pesticides to human health, their practices were poor. For example, one third of participants reported to have never used personal PPE during pesticides application. Knowledge about the name of the pesticides and knowledge about the use of gloves to reduce pesticide exposure had a significant association with the farmers’ attitudes towards safe use of pesticides. These findings appeal for the development of effective public health strategies to improve farmers’ awareness and provide information to develop integrated pest management.
